# A229 THE BACTERIAL VIRULENCE FACTOR NLEA UNDERGOES HOST-MEDIATED O-LINKED GLYCOSYLATION

**DOI:** 10.1093/jcag/gwac036.229

**Published:** 2023-03-07

**Authors:** L Burns, N Giannakopoulou, L Zhu, Y Z Xu, R H Khan, S Bekal, E Schurr, T M Schmeing, S Gruenheid

**Affiliations:** 1 Microbiology & Immunology; 2 Medicine, McGill University, Montreal; 3 Food Science and Agricultural Chemistry, McGill University, Sainte-Anne-de-Bellevue; 4 Microbiologie, Infectologie et Immunologie, Universite de Montreal, Montreal; 5 Institut National de Sante Publique du Quebec, Sainte-Anne-de-Bellevue; 6 Biochemistry, McGill University, Montreal, Canada

## Abstract

NOT PUBLISHED AT AUTHOR’S REQUEST

**Disclosure of Interest:**

None Declared

